# Inhibition of tribbles protein-1 attenuates radioresistance in human glioma cells

**DOI:** 10.1038/srep15961

**Published:** 2015-11-02

**Authors:** Bo Tang, Wei Wu, Qing Zhang, Yongjin Sun, Yifen Cui, Fei Wu, Xiaowei Wei, Guangying Qi, Xingsi Liang, Fang Tang, Yunqian Li, Wenhai Fan

**Affiliations:** 1 Department of Hepatobiliary Surgery, Guilin Medical University, Affiliated Hospital, and Laboratory of Liver Injury and Repair Molecular Medicine, Guilin Medical University, Guilin, 541001, China; 2 Department of Neurosurgery, The First Hospital of Jilin University, Changchun, 130021, China; 3 Affiliated Zhongshan Hospital of Dalian University, Dalian, 116001, China

## Abstract

Radiotherapy is one of the remedies in the treatment of glioma. The radioresistance is a major drawback, of which the mechanism is unclear. Tribble protein and histone deacetylase are involved in the cancer pathogenesis. This study aims to test a hypothesis that the histone deacetylase inhibitors attenuate the radioresistance in human glioma cells. In this study, human glioma cells were cultured. The cells were treated with irradiation with or without a histone deacetylase inhibitor, butyrate. Apoptosis of the glioma cells was assessed by flow cytometry. The results showed that human glioma cells expressed a low level of Trib1, which was significantly up regulated by exposure to small doses (2 Gy/day for 4 days) of irradiation. Trib1-deficient glioma cells showed an enhanced response to irradiation-induced apoptosis. Exposure to small doses of irradiation, Trib1 formed a complex with pHDAC1 (phosphor histone deacetylase-1) to inhibit p53 expression in glioma cells. The presence of HDAC1 inhibitor, butyrate or parthenolide, significantly enforced irradiation-induced glioma cell apoptosis. In conclusion, the Trib1 plays a critical role in the development of radioresistance of glioma cells. The data suggest that inhibition of Trib1 or HDAC1 has the potential to prevent or attenuate the radioresistance.

The malignant glioma is a cancer in the central nervous system. The therapeutic effect on this tumor is poor currently^1^. Radiotherapy is one of the major remedies in the treatment of glioma^2^. One of the major drawbacks of radiotherapy is the development of radioresistance of the tumor^3^. The underlying mechanism of radioresistance is to be further investigated.

To induce apoptosis in tumor cells is a major pathway of radiotherapy^4^. Apoptosis is a programmed cell death. It is a physiological event in general; but the process of apoptosis can be regulated by a number of biochemical events, which trigger the process of degradation to kill the cells; radiotherapy is one of them^5^. However, during radiotherapy, the apoptosis process in cancer cells may be attenuated to enable cancer cells to develop radioresistance; the underlying mechanism is to be further understood.

Tribbles are a family of proteins which have a variety of roles, including involvement in the control of the cell cycle in the fruit fly, was named after these fictional animals^6^. Trib1-3 are postulated to act as adaptor molecules to regulate and integrate a wide range of signaling pathways. Tribble 1 (Trib1, also named C8FW, SKIP1) is one of the mammalian orthologs of Tribbles. It is a pseudokinase, has a conserved motif, which is similar to the catalytic domain of a serine/threonine kinase, but lacks an ATP binding site or one of the conserved catalytic motifs essential for kinase activity^7^. Thus, Trib1 is considered as a scaffold protein or an adaptor protein to facilitate the degradation of their target proteins. In addition, over expression of Trib1 by prostate cancer^8^, leukemia^9^, is also found.

It is suggested that oral administration of probiotics improves the cancer symptoms and the life quality of cancer patients^10^. Probiotics also improve immunity in the body^11^. Whether probiotics can play a role in modulating the development of the radioresistance has not been investigated. Some probiotics, such as *Clostridium butyricum*, can secret butyrate. Butyrate is an inhibitor of histone deacetylase (HDAC); the latter is required in the regulation of gene transcription. It was reported that butyrate suppressed HDAC1 activities^12^. Based on the above information, we hypothesize that irradiation increases the expression of Trib1 and HDAC1 in glioma cells, which can be prevented by the presence of butyrate. Thus, we carried out this study. The results showed that exposure to irradiation markedly increased the expression of Trib1 in glioma cells, which was abolished by the presence of butyrate sodium. Butyrate inhibited the p53 expression in glioma cells and increased the irradiation-induced glioma cell apoptosis. The data suggest that administration of butyrate may prevent the development of radioprotention in glioma cells during radiotherapy.

## Results

### Irradiation up regulates Trib1 expression in human glioma cells

**Figure 1:**
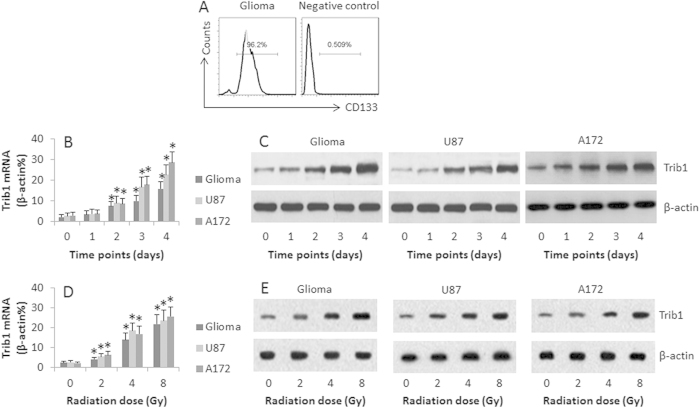
Irradiation up regulates Trib1 expression in glioma cells. Human glioma cells were isolated (By negative selecting method of MACS) from surgically removed glioma tissue (n = 10). (**A**) the isolated glioma cells were checked by flow cytometry. The histograms show the frequency of CD133 positive cells (a glioma marker). (**B**–**E**) the glioma cells, U87 cells and A127 cells were treated with radiation at 2 Gy/day for 4 consecutive days (**B**–**C**), or treated with graded doses (0–8 Gy) for 4 days (**D**–**E**). The cells were analyzed by RT-qPCR and Western blotting. The bars indicate the mRNA levels of Trib1 (**B**,**D**). The Western blots indicate the protein levels of Trib1 (**C**,**E**). The data are representatives of 10 (human samples) or 3 (cell lines) independent experiments. The data of bars are presented as mean ± SD. *p < 0.01, compared with the “0” group.

It is reported that Trib1 plays an important role in cancer cell activities^13^. Whether irradiation can regulate Trib1 expression in glioma cells is unclear. In the first step, we collected 10 human glioma specimens from surgically removed glioma tissue. The glioma cells were isolated and exposed to irradiation at gradient doses or the cells were exposed to one dose for different time. The cells were then analyzed by RT-qPCR and Western blotting. The results showed that the Trib1 was detected in the human glioma cells, which was markedly up regulated by exposing to low doses (2 Gy/day for 1–4 days, or at graded doses for 4 days) irradiation in a time-dependent manner (Fig. 1A,B) and in an irradiation dose-dependent manner (Fig. 1C,D). Such a feature was also detected in glioma cell lines, the U87 cells and A172 cells (Fig. 1A,D). In addition, we also detected the expression of Trib2 and Trib3 in the glioma cells, which was not altered by irradiation (data not shown). The data indicate that human glioma cells express Trib1, which can be up regulated by irradiation, which may be associated with glioma cell activities in a radiation environment.

### Trib1 interferes with irradiation-induced apoptosis in human glioma cells

**Figure 2:**
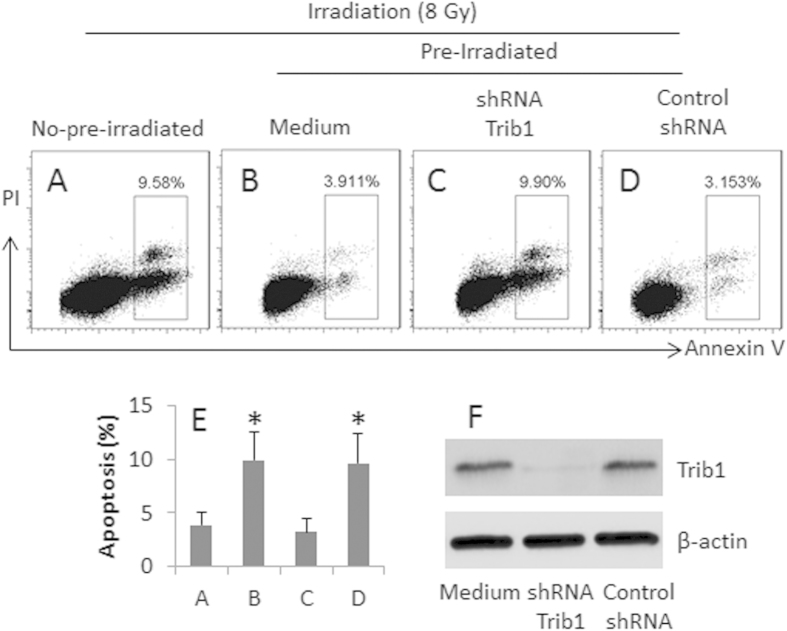
Trib1 interferes with irradiation-induced U87 cell apoptosis. U87 cells were pre-irradiated and re-irradiated at a large dose (8 Gy). The cells were analyzed by flow cytometry 24 h later. (**A**–**D**) the gated dot plots show the frequency of apoptotic cells. (**E**) the bars show the summarized data of (**A**–**D)**. (**F**) the Western blots show the Trib1 RNAi results. The data are representatives of 3 independent experiments.

Exposure to low doses of radiation may induce radioresistance in cancer cells; the mechanism has not been fully elucidated. We next investigated the association of Trib1 with the development of radioresistance in glioma cells. U87 cells were treated with or without a 4-day low dose irradiation-sensitization; the sensitized cells and control cells were treated with irradiation at 8 Gy; the cells were then analyzed by flow cytometry. The results showed that the frequency of apoptotic cells in the sensitized group was significantly lower than control group (Fig. 2). The data suggest that pretreatment with irradiation prevents U87 cells from the re-irradiation-induced apoptosis. Since the treatment with low doses of radiation induced high levels of Trib1 in U87 cells as shown by Fig. 1, we inferred that the Trib1 might interfere with irradiation-induced U87 cell apoptosis. To test the hypothesis, we knocked down the Trib1 gene in U87 cells by RNAi, then exposed the cells to irradiation. The results showed that the Trib1-RNAi markedly increased the irradiation-induced apoptosis as compared with controls (Fig. 2).

### Trib1 mediates irradiation-induced HDAC1 phosphorylation in U87 cells

**Figure 3:**
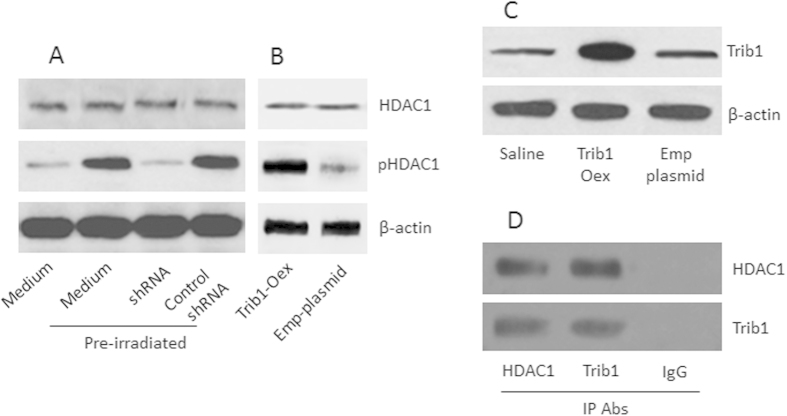
Trib1 is required in the pre-irradiation-induced HDAC1 phosphorylation in U87 cells. Trib1-sufficient or –deficient U87 cells were treated with or without pre-irradiation. (**A**) the Western blots show the protein levels of HDAC1 and pHDAC1. shRNA: Trib1 shRNA. (**B**) U87 cells were over-expressed (Oex) Trib1. The immune blots indicate the levels of HDAC1 in the U87 extracts. Emp-plasmid: U87 cells were transfected with empty plasmids using as a negative control. (**C**) the Western blots indicate the results of Trib1 over-expression in U87 cells. (**D**) the Western blots show the Trib1/pHDAC1 complexes (by immunoprecipitation). The data are representatives of 3 independent experiments.

It is reported that HDAC1 is associated with the development of radioresistance in cancer cells^14^. We wondered if Trib1 contributed to the HDAC1 phosphorylation in glioma cells. To this end, we firstly measured the HDAC1 phosphorylation in U87 cells. The results showed that the pre-treatment with low dose irradiation induced HDAC1 phosphorylation in U87 cells. To test if Trib1 is required in the HDAC1 phosphorylation in the U87 cells, we knocked down the Trib1 gene in U87 cells, then treated the cells with irradiation. Indeed, the induction of HDAC1 phosphorylation was abolished (Fig. 3A). The results indicate that Trib1 mediates the irradiation-induced HDAC1 phosphorylation in U87 cells. To enforce the results, U87 cells were over-expressed with Trib1 (Fig. 3C), which significantly increased the levels of the pHDAC1 in the U87 cells (Fig. 3A).

### Trib1 forms a complex with HDAC1 to repress p53 transcription

**Figure 4:**
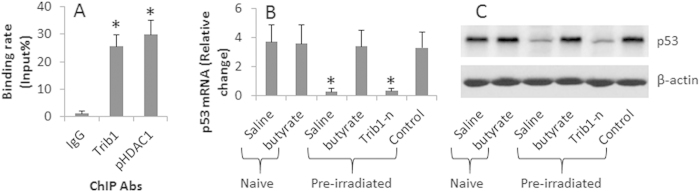
Trib1/pHDAC1 complexes modulate p53 expression in U87 cells after pre-irradiation. U87 cells were treated with pre-irradiation. (**A**) the bars show the binding rate of the p53 promoter by Trib1/pHDAC1 complexes (analyzed by ChIP). (**B**) the bars show the mRNA levels of p53 in U87 cells after treatments as denoted on the X axis. (**C**) the Western blots show the protein levels of p53 in U87 cells. Butyrate = 5 μM. Trib1-n: Trib1-deficient U87 cells. Control: U87 cells were treated with control shRNA. The data are representatives of 3 independent experiments.

Published data indicate that the HDAC1 suppresses p53 in solid tumors^15^. We wondered if the irradiation-induced HDAC1 activation also regulated p53 in glioma cells. To this end, after pre-irradiation, we extracted proteins from U87 cells and analyzed by immunoprecipitation. The results showed that a complex of Trib1 and pHDAC1 was detected in the U87 cells (Fig. 3D). Further analysis showed that the complex of Trib1 and pHDAC1 bound to the p53 promoter (Fig. 4A) and repressed the expression of p53 in U87 cells (Fig. 4B,C). The results suggest that exposure to low doses of irradiation inhibits p53 expression in U87 cells, in which the expression of Trib1 and activation of HDAC1 are involved.

### Blocking HDAC1 prevents development of radioresistance in U87 cells

**Figure 5:**
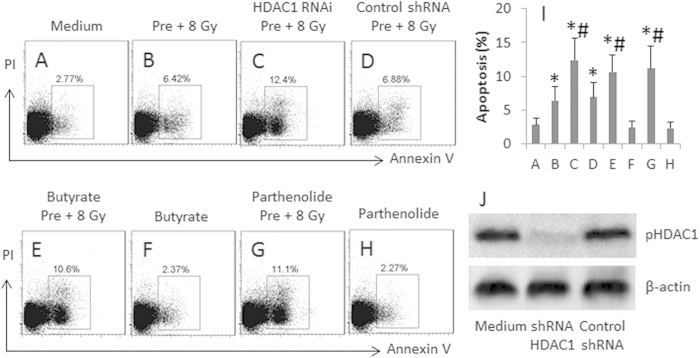
Inhibition of HDAC1 attenuates irradiation-induced radioprotection in U87 cells. The treatment of U87 cells is denoted above each dot plot panel. Pre: Pre-irradiation. 8 Gy: Re-irradiation at 8 Gy. Butyrate = 5 μM. Parthenolide = 10 μM. The data are representatives of 3 independent experiments. (**A-H**), the gated dot plots show the frequency of apoptotic U87 cells. (**I**), the bars indicate the summarized data of (**A-H**). (**J**), the Western blots show the HDAC1 RNAi results. The data of bars are presented as mean ± SD. *p < 0.05, compared with group A. ^#^p < 0.05, compared with group B. The data are representatives of 3 independent experiments.

The above results implicate that HDAC1 plays an important role in the development of radioresistance in U87 cells. To enforce the results, we knocked down the HDAC1 gene in U87 cells; the HDAC1-deficient U87 cells were treated with pre-irradiation and re-irradiation. The cells were analyzed by flow cytometry. The results showed that the frequency of apoptotic cell was significantly less in the HDAC1-deficient U87 cells as compared with HDAC1-sufficient U87 cells. On the other hand, we added HDAC1 inhibitor, butyrate sodium or parthenolide, to the culture medium of U87 cells during the pre-irradiation and re-irradiation, which also abolished the development of radioresistance in the U87 cells (Fig. 5). The results support that HDAC1 plays an important role in the development of radioresistance in U87 cells.

## Discussion

The data showed that after repeated exposures to small doses of irradiation, the apoptotic glioma cells were reduced markedly as compared to control glioma cells, accompanying the increases in HDAC1 phosphorylation, in which Trib1 was required. The Trib1/HDAC1 complexes bound to the p53 promoter to interfere with the expression of p53 in glioma cells. The data have revealed that Trib1 plays a critical role in the development of radioresistance in glioma cells.

The development of radioresistance is a biological reaction of biological cells. Many factors have been identified in the development of radioresistance. For example, HIF1α over expression can be a biomarker for radioresistance in prostate cancer^16^. The Kelch-like ECH-associated protein 1 (Keap1)-nuclear factor E2-related factor 2 (Nrf2) pathway is one of the major signaling cascades involved in cell defense and survival against radiation via metabolic reprogramming and inhibition of apoptosis^17^. Our data reveal a new member of the molecular pool of radioresistance inducer, the Trib1, which plays a critical role in the radioresistance development in glioma cells in the repeated exposures to small doses of radiation.

Previous studies showed that Trib1 was associated with the pathogenesis of cancer. It is suggested that Tribs are central signaling mediators in different subtypes of acute leukemia and proposes that inhibition of dysregulated Trib signaling may be therapeutically beneficial^18^. Lu *et al.* indicate that the *TRIB1* gene is associated with pancreatic cancer^8^. Tribble proteins are also involved in a series of non-neoplastic disorders, including metabolic and neurological diseases^7^. In line with those published data, our results suggest that Trib1 is also involved in the pathogenesis of cancer by conferring glioma cells the ability of radioresistance.

The development of radioresistance is a large drawback of radiotherapy. Thus, to inhibit or attenuate radioresistance has the potential to promote the therapeutic efficiency of radiotherapy. Steglich reported that simultaneous α3 and β1 integrin inhibition led to higher cytotoxicity and reduced the radioresistance in head and neck squamous cell carcinoma^19^. Brett-Morris reported that SAT1 (spermidine/spermine-N1-acetyltransferase 1) promoted resistance to ionizing radiation (IR) in glioma that contributed to glioma cell radioresistance, and thus suggests that SAT1 may potentially be a therapeutic target to sensitize GBM to cancer therapies^20^. Similar to those previous studies, we identified that Trib1 played a critical role in the radioresistance of glioma cells. Furthermore, we found that interference with the signal transduction pathway of the Trib1-induced radioresistance in glioma cells resulted significant enhancement of the radiosenstive of the cells.

Our data show that the presence of butyrate sodium significantly enhanced the sensitivity of glioma cells to irradiation. Such an effect may be because the Trib1 forms a complex with HDAC1; the complex binds to the p53 promoter to inhibit p53 expression in glioma cells as shown by the present data. The data suggest that the activation of HDAC1 is a checkpoint of the development of radioresistance in glioma cells. Since butyrate is an inhibitor of HDAC1, the presence of butyrate in the prevention of radioresistance in glioma cells should be the effect of HDAC1-inhibition. The inference is supported by the presence of parthenolide, the specific inhibitor of HDAC1.

## Materials and Methods

### Reagents

The antibodies of Trib1, HDAC1, pHDAC1, p53, and shRNA kits of Trib1 and HDAC1 were from Santa Cruz Biotech (Shanghai, China). The Annexin V kit, protein G, butyrate sodium and Chip kit were from Sigma Aldrich (Shanghai, China). The reagents for RT-qPCR and Western blotting were from Invitrogen (Shanghai, China).

### Cell culture

Human glioma cell lines, U87 cells and A172 cells were purchased from ATCC (Beijing, China). The cells were cultured in Dulbecco’s Modified Eagle Medium supplemented with 10% fetal bovine serum, 100 U/ml penicillin, 0.1 mg/ml streptomycin and 2 mM L-glutamine. The cell viability was greater than 98% before using for further experiments as assessed by Trypan blue exclusion assay.

### Irradiation

Cells were exposed to irradiation from a Gammacell 40 137Cs irradiator (dose rate, 1.0 Gy/min) at the Guilin Medical University (Guilin, China). The pre-irradiation was performed on the cells at 2 Gy/day, or treated with graded doses of radiation, for 4 consecutive days. The irradiation of the cells was performed within 8 min.

### Patients

Nine glioma patients (Male = 5; female = 4; age = 16 ~ 46 years with a mean of 26.6 years old) were recruited into the present study. The diagnosis and treatment of glioma were performed by their surgeons at the Affiliated Hospital of Guilin Medical University. The using human tissue in the present study was approved by the Human Ethic Committee at Guilin Medical University. An informed, written consent was obtained from each subject. The methods were carried out in “accordance” with the approved guidelines.

### Preparation of single glioma cells

The surgically removed glioma tissue was collected from surgical operation rooms. The tissue was cut into small pieces, incubated with collagenase IV (1.0 mg/ml) at 37 °C for 2 h with mild agitation. The cells were filtered with a cell strainer (40 μm). The immune cells (T cells, B cells, CD14^+^ cells and dendritic cells) and fiberocytes were isolated by the magnetic cell sorting (MACS) with proper reagent kits following the manufacturer’s instructions. The single glioma cells were cultured in DMEM. The culture medium was supplemented with 100 U/ml penicillin, 10% fetal bovine serum, 0.1 mg/ml streptomycin and 2 mM glutamine. The cell viability was assessed by Trypan exclusion assay.

### Apoptosis analysis by flow cytometry

Apoptosis were performed by costaining the cells with propidium iodide (PI) and fluorescein isothiocyanate (FITC)-Annexin V conjugates following the manufacturer’s instructions.

### Quantitative real time -PCR (qRT-PCR)

Total RNA was extracted from cell samples with TRIzol reagents, cDNA was synthesized with a reverse transcription kit. The qPCR was performed on the CFX96 Touch™ Real-Time PCR Detection System (Bio Rad) with SYBR Green Master Mix. The samples were normalized to the β-actin mRNA and were calculated by the 2^−ΔΔCT^ method. The primers using in the present study include: Trib1, forward, ccaagatgctgcagaccatc; reverse, ggccgatatgtgactaggct. P53, forward, tggccatctacaagcagtca; reverse, ggtacagtcagagccaacct.

### Small RNA interference

The cells were treated with shRNA of Trib1 or HDAC1 following the manufacturer’s instructions. The effect of gene knockdown was assessed by Western blotting 48 h after the transduction.

### Immunoprecipitation (IP) and immunoblotting

Protein extracts were incubated overnight at 4 °C with anti-Trib1 (1 μg/ml) or anti-pHDAC1 (1 μg/ml), and non-specific rabbit IgG (1 μg/ml; using as a control), respectively. Samples were incubated with protein G Sepharose beads for 3 h at 4 °C. The immunoprecipitated proteins were eluted from the beads with SDS buffer and immunoblotted using the indicated primary antibodies. Briefly, the proteins were fractioned by SDS-PAGE (sodium dodecyl sulfate polyacrylamide gel electrophoresis) and transferred onto a PVDF membrane. After blocking with 5% skim milk for 30 min at room temperature, the membrane was incubated with antibodies of interest overnight at 4 °C and followed by incubating with the secondary antibodies (labeled by peroxidase) for 1 h at room temperature. Washing with TBST (Triss-buffered saline Tween 20) was performed after each incubation. The immunoblots on the membrane were developed with ECL (enhanced chemiluminescence). The results were photographed with an image system (UVI, Beijing, China).

### Chromatin immunoprecipitation (ChIP)

The cells were fixed in 1% formaldehyde to cross link protein and DNA, and lysed. The fixed lysates were sonicated and precleared with Dynabeads Protein A (Invitrogen, Shanghai, China) and immunoprecipitated overnight at 4 °C with antibodies against pHDAC1 or Trib1 or non-specific IgG. The samples were treated with Proteinase K (45 °C for 2 h) in a buffered salt solution. DNA was purified with a ChIP purification kit (Sigma Aldrich, Shanghai, China). Primers (tcctccccaactccatttcc; ggacggtggctctagacttt) of p53 promoter sites were used to assess the binding rate of the complex of Trib1/pHDAC1 via qPCR. The results were normalized to the input control sample (sheared chromatin collected before antibody addition, equivalent to 1% of the IP sample volume).

### Over expression of Trib1 in U87 cells

A plasmid carrying the Trib1 gene was provided by Genescript (Nanjing, China). U87 cells were transfected with the Trib1-carrying plasmid or an empty plasmid following the manufacturer’s instructions. The Trib1 gene over expression by the U87 cells was checked by Western blotting.

### Statistics

The data are presented as mean ±SD. Differences between groups were determined with the Student t test or ANOVA if more than two groups. A p < 0.05 was set as a significant criterion.

## Additional Information

**How to cite this article**: Tang, B. *et al.* Inhibition of tribbles protein-1 attenuates radioresistance in human glioma cells. *Sci. Rep.* **5**, 15961; doi: 10.1038/srep15961 (2015).
